# Ion Clusters Reveal the Sources, Impacts, and Drivers of Freshwater Salinization

**DOI:** 10.1021/acs.est.5c04512

**Published:** 2025-06-16

**Authors:** Diver E. Marin, Stanley B. Grant, Shantanu V. Bhide, Megan A. Rippy, Jesus D. Gomez-Velez, Robert N. Brent, Sujay S. Kaushal, Harold Post, Sydney Shelton, Shalini Misra, Erin R. Hotchkiss, Ahmed Monofy, Dongmei Alvi, Bradley Schmitz, Shannon Curtis, Christina C. Davis, Peter Vikesland, Admin Husic

**Affiliations:** † Occoquan Watershed Monitoring Laboratory, Department of Civil and Environmental Engineering, Virginia Tech, Manassas, Virginia 20110, United States; ‡ Environ. Sci. Division, Oak Ridge National Laboratory, Oak Ridge, Tennessee 37830, United States; § School of Integrated Sciences, James Madison University, Harrisonburg, Virginia 22807, United States; ∥ Department of Geology and Earth System Science Interdisciplinary Center, University of Maryland, College Park, Maryland 20742, United States; ⊥ School of Public and International Affairs, Virginia Tech, Arlington, Virginia 22203, United States; # Department of Biological Sciences, Virginia Tech, Blacksburg, Virginia 24061, United States; ∇ Loudoun Water, Ashburn, Virginia 20147, United States; ○ Fairfax County Public Works and Environmental Services, Fairfax, Virginia 22035, United States; ◆ Dept. of Civil and Environ. Engineering, Virginia Tech, Blacksburg, Virginia 24061, United States

**Keywords:** salinization, streams, urbanization, stormwater, ecology, groundwater, deicers

## Abstract

Population growth, land use change, climate change, and natural resource extraction are driving the salinization of freshwater resources worldwide. Reversing these trends will require data-centric approaches that identify salt sources, environmental drivers, and ecosystem responses. In this study, we applied principal component analysis and hierarchical clustering to identify ion covariance patterns, or “ion clusters,” in Broad Run, an urban stream in the Mid-Atlantic United States. These clusters correspond to distinct hydrologic regimes and reveal specific salinization risks: (1) phosphorus pollution mobilized during summer storms (Cluster 1); (2) elevated concentrations of sulfate and bicarbonate during baseflow (Cluster 2), likely reflecting groundwater discharge; and (3) elevated specific conductance and sodium, chloride, and potassium ion concentrations during snowmelt and rain-on-snow events (Cluster 3), driven by deicer and anti-icer wash-off. These ion fingerprints offer a transferable framework for diagnosing salt sources, assessing ecological risk, and identifying management targets. Our findings underscore the need for next-generation stormwater infrastructure and smart growth policies to protect aquatic life in rapidly urbanizing watersheds.

## Graphical abstract

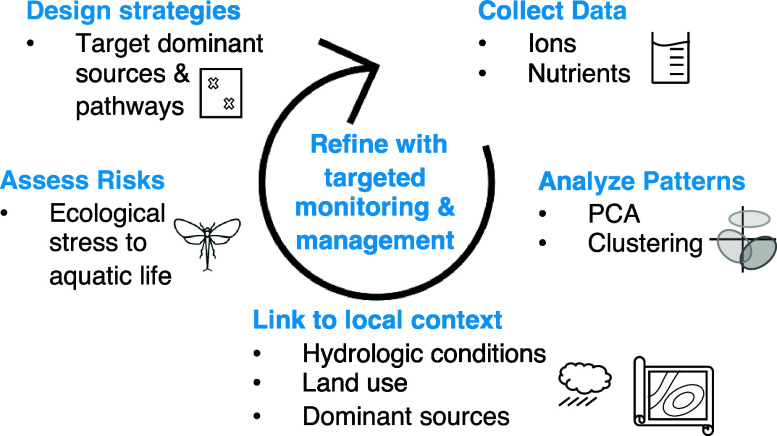

## Introduction

Dissolved ions occur naturally in freshwater systems due to atmospheric deposition^1^ and the weathering of soils and bedrock.^2^ Yet across the globe, salinity levels in rivers, streams, lakes, and groundwater are rising due to human activities.^3^ In temperate urban and suburban areas, these changes are driven by overlapping inputs from road deicers, construction materials, fertilizers, storm runoff, and treated wastewater.^3–8^ As a result, ion concentrations are increasing even in regions without historic salinization issues,^9^ prompting concern about ecological degradation and long-term sustainability.^10^

Salinization can reduce freshwater biodiversity and ecosystem function^11–13^ with impacts ranging from osmotic stress for sensitive species to the mobilization of legacy pollutants.^14–17^ While regulatory efforts have historically focused on single-ion thresholds (e.g., chloride^18^) or aggregate metrics (e.g., specific conductance^19^), emerging research highlights the importance of ion mixtures and their interactions.^20,21^ These mixtures can differ substantially across space and time depending on the source, transport pathway, and hydrologic context.^22^

In this study, we investigate patterns of stream salinization in Broad Run, a rapidly urbanized watershed in Northern Virginia. Using 3 years of stream monitoring data, we apply principal component analysis (PCA) and hierarchical clustering to identify groups of samples with similar ion covariance patterns, which we refer to as *ion clusters*. We interpret these clusters with respect to seasonal hydrologic conditions and use them to evaluate potential sources, ecological risks, and management strategies.

This study is novel in its use of ion clustering as an organizing framework for diagnosing salinity drivers and their impacts on stream ecosystems. We hypothesize that distinct ion mixtures reflect not only salt source types but also transport mechanisms and retention times, which vary seasonally and across flow regimes. Specifically, we ask: (1) What are the statistically distinct ion mixtures in this urban stream, and how do they relate to hydrologic conditions? (2) What do these mixtures reveal about the likely sources and pathways of salinity inputs? (3) How do ion mixtures relate to aquatic life stress, as inferred from regional bioassessment thresholds? By addressing these questions, we demonstrate how statistical pattern recognition tools can reveal actionable insights for managing freshwater salinization in urbanizing watersheds.

## Materials and Methods

### Site Description

Our monitoring station (BL30; 39.024°N, 77.439°W) is located on a fourth-order reach of Broad Run in the Potomac-Shenandoah River Basin (Figure S1 and Note S1). This portion of Broad Run drains a rapidly urbanizing 152 km^2^ catchment in Loudoun County, Virginia, USA. Impervious cover in this subwatershed increased from approximately 19% in 2001 to approximately 32% in 2019.^23^ The drainage lies within the Mesozoic Lowlands hydrogeomorphic region, underlain by siltstone, shale, sandstone, diabase, and basaltic rock types^24,25^ (Figures S2 and S3). There are no wastewater treatment plant discharges upstream of BL30.

### Sample Collection and Field Measurements

Baseflow and storm samples were collected at BL30 over a three-year period (2020-04-13 to 2023-05-03). Biweekly grab samples were collected to represent the relatively stable conditions of baseflow, whereas flow-weighted composite samples were used to capture representative pollutant concentrations during storm events, when concentrations can vary considerably over the storm hydrograph.^26^ All water samples were transported on ice to the Occoquan Watershed Monitoring Laboratory either immediately (baseflow) or within 24 h (storm samples) of collection. Field measurements of streamwater temperature, pH, specific conductance (SC), and total alkalinity were conducted during baseflow sampling. For safety reasons, no field measurements were conducted during storm events (Note S2).

### Laboratory Analysis

#### Dissolved Ions

Filtered baseflow and storm samples were analyzed within 1 day of arrival at the laboratory for major dissolved ions (K^+^, Na^+^, Cl^–^, SO_4_^2–^, Ca^2+^, Mg^2+^) using ion chromatography (Dionex ICS-5000) following ASTM D6919-09 and SM 4110 B-2011. In baseflow samples, bicarbonate concentrations were calculated from field-measured alkalinity (Notes S3 and S4).

#### Nutrients

Unfiltered samples were analyzed for total nitrogen (TN) and total phosphorus (TP) following persulfate digestion. Filtered subsamples were analyzed for dissolved nitrate + nitrite (NO_3_^–^/NO_2_^–^) and orthophosphate (PO_4_^3–^) using Astoria Pacific autoanalyzers and Standard Methods 4500-P J-2011, 4500-NO_3_^–^ F-2011, and 4500-P F-2011. Additional details, including sample preservation, instrumentation, and detection limits, are provided in Note S3 and Table S3.

#### Total Suspended Solids

Total suspended solids (TSS) were measured gravimetrically, following Standard Method 2540 D-2011.

### Left-Censored Values and Charge Balance Error (CBE)

All left-censored values (i.e., values below the limit of detection (LOD), Table S3) were set equal to one-half the LOD. Charge balance error (CBE) was calculated for each sample based on the normality of all measured or imputed cations (K^+^, Na^+^, Ca^2+^, Mg^2+^, NH_4_^+^, H^+^) and anions (HCO_3_^–^, Cl^–^, SO_4_^2–^, NO_3_^–^/NO_2_^–^, PO_4_^3–^) as described in Note S5.

### Imputation of Missing Data

Missing values were imputed using regularized iterative Principal Component Analysis (PCA) via the estim_ncp and imputePCA functions in the MissMDA package (R, version 4.4.1). The estim_ncp function estimates the optimal number of principal components which are then used by imputePCA to perform the imputation.^27^ Imputed bicarbonate concentrations in storm samples were validated using two approaches: (1) comparison to bicarbonate concentrations estimated via a geochemical charge-balance method, in which all unbalanced anionic charge is attributed to bicarbonate (Note S4); and (2) evaluation of CBE calculated from the measured and imputed ion concentrations for each storm sample (Note S5).

### Environmental Variables

#### Stream Discharge

Stream discharge was estimated from hourly stage measurements collected with a pressure transducer (Pressure Systems, Inc., 10 PSI Transducers) fixed to a wooden monument near the stream bank, just upstream of a stable cross-section provided by a sewer line crossing. Stage measurements were converted to discharge, stored in a data logger (Sutron 8210), and retrieved during baseflow sample collection. Hourly stream discharge measurements were averaged to daily discharge for further analysis (Note S6). The stage-discharge relationship was updated every 3 to 6 months (and after large storms) following USGS Method 3-A8.^28^

#### Baseflow Index (BFI)

Daily estimates of baseflow, *Q*_BF_, were computed by applying a Recursive Digital Filter to the measured discharge data, *Q* (grwat package in R Software, version 4.4.1, R Core Team 2024)^29,30^ (Note S7). The baseflow index was calculated as BFI = *Q*_BF_/*Q*.

#### Precipitation, Air Temperature, and Snow Melt

Hourly precipitation and air temperature data were collected from the weather station at Dulles International Airport (located within the drainage area, 10 km to the south of BL30) (Note S6). Snowmelt was estimated using the Hydrologiska Byråns Vattenbalansavdelning (HBV) model^31,32^ (Note S8).

#### Local Groundwater Data

Groundwater data were obtained from the U.S. Geological Survey’s National Water-Quality Assessment (NAWQA) program.^33^ Specifically, we accessed records from USGS well no. 385930077215901, located approximately 7 km southeast of BL30 (Figure S2). Between 2003 and 2015, the USGS collected eight samples from this well (at depths of 20–90 feet below ground surface) and analyzed them for the same suite of inorganic ions and nutrients described above for Broad Run.

### Principal Component Analysis (PCA) and Hierarchical Clustering

Prior to PCA, ion and nutrient concentrations were log-transformed to reduce skewness and standardized (*Z*-scored). The number of significant principal components (PCs) was determined using a resampling-based stopping rule, which identifies components that explain more variance than expected by chance (*p* < 0.05)^34,35^ (Figure S8 and Note S9).

Hierarchical clustering was then applied in principal component space using Euclidean distance and the Ward criterion, as implemented in the HCPC function from the FactoMineR package (R version 4.4.1).^36^ The optimal number of clusters was identified based on the relative loss of inertia (i.e., within-cluster variance), with the selected partition corresponding to the point at which additional clusters result in a substantially smaller gain in explanatory power. Permutational multivariate analysis of variance (perMANOVA) with Euclidean distance was used to test for statistically significant differences among the identified clusters.^37^ As a visual guide, ellipses were drawn to represent the approximate region of principal component space occupied by each cluster; specifically, each ellipse represents the region of principal component space that would be expected to include 90% of samples associated with a particular cluster, assuming a multivariate normal distribution of the samples within each cluster.

### Median Comparison Tests

Nonparametric bootstrapping was used to test for significant differences in group medians (using the boot package in R version 4.4.1).^38^ Significance was assessed at the *p* < 0.05 level using bias-corrected and accelerated confidence intervals, with adjustments for multiple comparisons made using the Bonferroni correction.^39^

### Benthic Macroinvertebrate Responses

Since 2001, the Virginia Department of Environmental Quality (VDEQ) has collected stream ion concentrations and biological integrity metrics—including the Virginia Stream Condition Index^40^—at 473 randomly selected monitoring stations across the state as part of its probability-based monitoring program.^41^ To contextualize the measurements at BL30, we compared them to (1) concentrations measured at the VDEQ probability-based monitoring sites within the Northern Piedmont Ecoregion (for ecoregion-specific percentiles) and (2) statewide aquatic life stress thresholds established by the VDEQ, which are currently not available at a finer ecoregional resolution (see Note S10 for details).

First, for each ion and nutrient measurement at BL30, we calculated its percentile ranking relative to concentrations measured at VDEQ monitoring sites within the Northern Piedmont Ecoregion, where BL30 is located, on the premise that stream stations within the same ecoregion will support similar macroinvertebrate assemblages and exhibit responses similar to those of environmental stressors.

Second, we compared the ion and nutrient concentrations at BL30 to statewide benthic community response thresholds developed by VDEQ.^41^ These thresholds, derived from the agency’s probabilistic monitoring data set using quantile regression and conditional probability analyses,^42^ define concentration ranges associated with increasing probability of stress to aquatic life across all Virginia ecoregions: “No Probable Stress” (background conditions), “Low Probability of Stress” (minor biological response), “Medium Probability of Stress” (substantial biological response), and “High Probability of Stress” (significant degradation of the benthic community).

## Results

### Stream Flow

Stream discharge at BL30 ranged from 0.113 to 4.94 mm/day during baseflow sampling (median = 0.310 mm/day) and from 1.59 to 22.0 mm/day during storm sampling (median= 5.48 mm/day).

### Water Quality Data Set

A balanced number of baseflow (*N* = 79) and storm (*N* = 74) samples were collected at BL30 over the three-year study period. After the imputation of missing values, four samples (two baseflow and two storm) had an absolute charge balance error (CBE) >10% and were excluded from further analysis (Note S5). The remaining samples (*N* = 149) had CBEs ranging from −4.37% to +6.23% (baseflow samples, *N* = 77) and from −4.2% to +7.01% (storm samples, *N* = 72).

Ion concentrations were generally above their lower limits of detection (LODs). The exceptions were NH_4_^+^ and PO_4_^3–^, which were below their respective LODs in 2 and 27 samples, respectively.

The final data set included 2,086 measured and 179 imputed values for specific conductance (SC), total phosphorus (TP), total nitrogen (TN), total suspended solids (TSS), and dissolved inorganic cations (Ca^2+^, Mg^2+^, NH_4_^+^, K^+^, Na^+^, H^+^) and anions (Cl^–^, SO_4_^2–^, NO_3_^–^/NO_2_^–^, PO_4_^3–^, HCO_3_^–^).

### Principal Component Analysis (PCA)

PCA was performed on measured or imputed concentrations of cations (Ca^2+^, Mg^2+^, K^+^, Na^+^, NH_4_^+^, H^+^) and anions (Cl^–^, SO_4_^2–^, NO_3_^–^/NO_2_^–^, HCO_3_^–^); because a relatively large number of PO_4_^3–^ measurements were left censored (see last section), this ion was not included in the PCA. The goal of the PCA was to identify statistically distinct ion covariance patterns, and therefore bulk nutrients (TP and TN) and TSS measurements were also not included in the analysis.

Two significant principal components explained 73% of the total variance in ion measurements (Figure 1). The first principal component (PC1) reflects overall ion concentration, with samples scoring positively along PC1 characterized by elevated ion levels (gray arrows in the figure). The second principal component (PC2) differentiates between samples enriched in monovalent ions (positive PC2) and samples enriched in divalent ions and carbonate system constituents (negative PC2).

**Figure 1:**
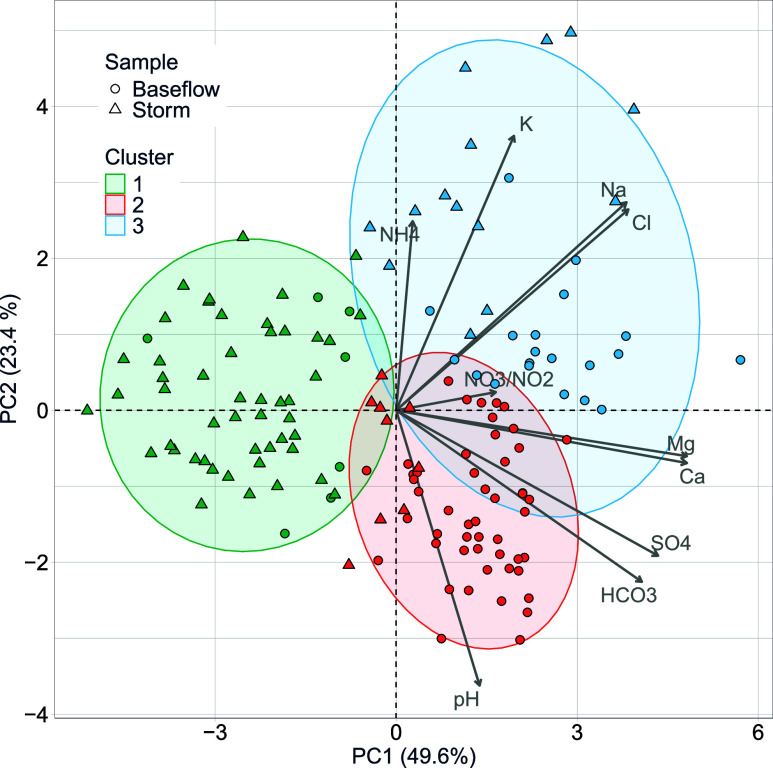
73% of the variance in ion concentrations measured at Broad Run is captured by the two principal components PC1 (49.6%) and PC2 (23.4%). The position in biplot space of all *N* = 77 baseflow samples (circles) and *N* = 72 storm samples (triangles) are shown, along with loadings for the ions included in the PCA (Ca^2+^, Mg^2+^, NH_4_^+^, K^+^, Na^+^, pH, Cl^–^, SO_4_^2–^, NO_3_^–^/NO_2_^–^, HCO_3_^–^). The colored ellipses are included here only as a visual aid; the actual boundary between clusters is not elliptical. Importantly, despite some overlap between ellipses, the hierarchical clustering method assigned each baseflow and storm sample to one of three clusters (Cluster 1, 2, or 3). Results from the perMANOVA test indicate that the three clusters are significantly different (*p* < 0.05).

### Hierarchical Cluster Analysis

Hierarchical clustering in principal component space identified three statistically distinct ion covariance patterns, or ion clusters, associated with the samples collected at BL30 (Figure 1). As documented below, these three ion clusters correspond to (1) summer storm events (Cluster 1), (2) summer and winter baseflow conditions (Cluster 2), and (3) winter snowmelt and rain-on-snow events (Cluster 3).

#### Environmental Drivers

Cluster 1 is primarily composed of samples collected during summer storm events, as indicated by the high proportion of storm samples, elevated median air temperature and rainfall, and a baseflow index (BFI) near zero (Figure 2a).

**Figure 2:**
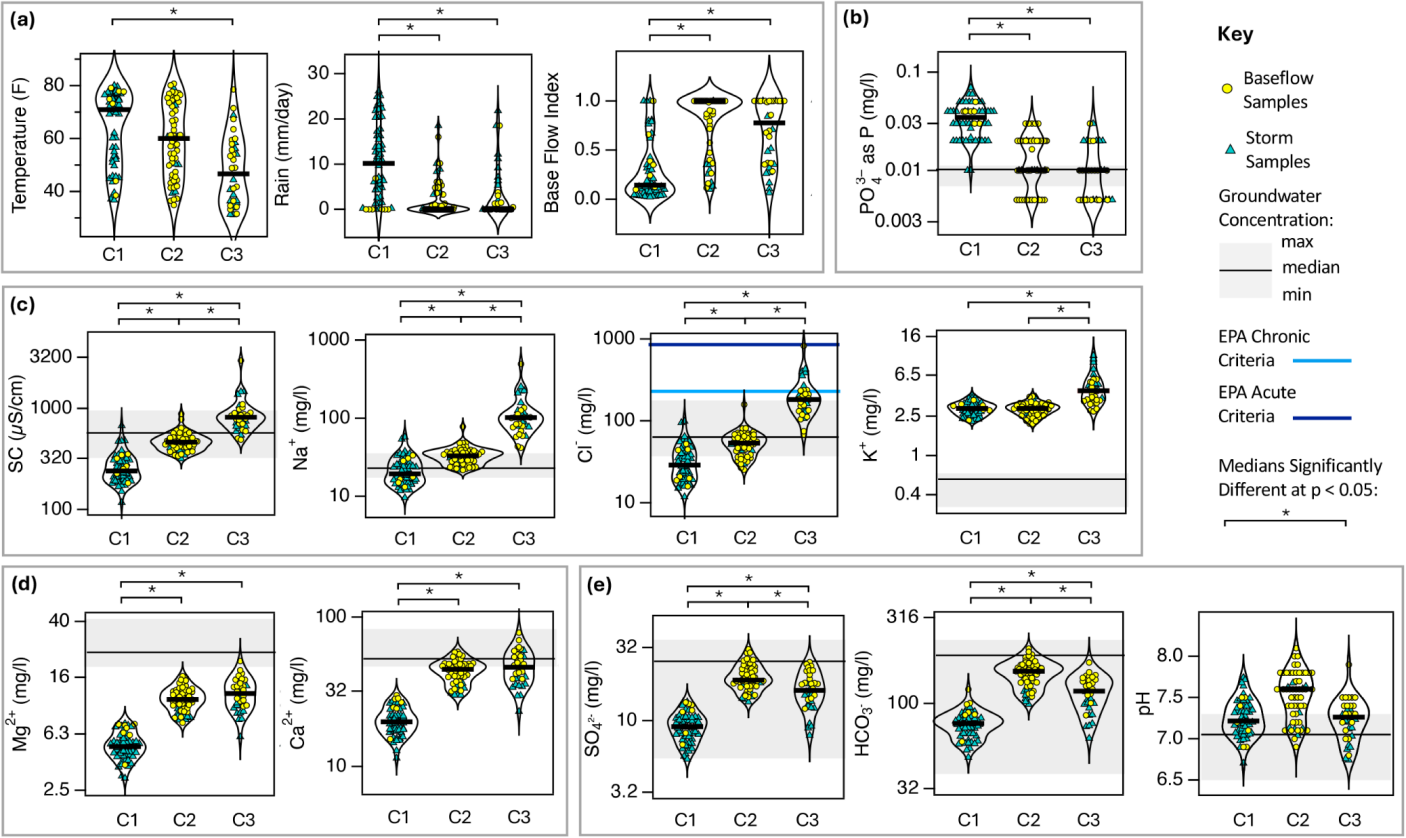
Violin plots of the (a) environmental drivers and (b)–(e) ion concentrations associated with each of the three clusters (C1 = Cluster 1 (summer storms), C2 = Cluster 2 (baseflow), and C3 = Cluster 3 (snowmelt and rain-on-snow events)). Ion concentrations are divided into those for which the median concentrations are highest in (b) C1, (c) C3, (d) C2 and C3, or (e) C2. The median concentrations for each ion are indicated by a thick black horizontal line. Baseflow and storm samples are indicated by yellow circles and teal triangles, respectively. Any pair of median values are significantly different (at *p* < 0.05) when connected by starred brackets. Thin black horizontal line and gray region denotes the median and range of ion concentrations measured in a nearby groundwater well. Orthophosphate concentrations below the limit of detection (LOD) of 0.01 mg/L were set to one-half the LOD. Values of pH, SC, and HCO_3_^–^ in storm samples were imputed.

Cluster 2 is primarily composed of summer and winter baseflow samples, characterized by a median BFI near unity, low median rainfall, and a wide range of air temperatures (Figure 2a).

Cluster 3 is primarily composed of samples collected during winter snowmelt or rain-on-snow events—a common condition in this region. These samples are marked by low median air temperatures, low median rainfall, intermediate median BFI, and timing consistent with snowmelt predictions from the HBV model (10 of 31 Cluster 3 samples occurred during modeled snowmelt periods; data not shown) (Figure 2a).

#### Geochemical Signatures

Samples in Cluster 1 are characterized by relatively low median concentrations of most ions, consistent with the dilution by urban runoff during summer storm events (Figure 2c–e). Two exceptions are orthophosphate (PO_4_^3–^) and potassium (K^+^) ion concentrations (Figure 2b,c). Potassium ion concentrations are particularly elevated during winter deicing events (Cluster 3) but remain high (relative to groundwater) during both baseflow conditions and summer storms (Clusters 2 and 1). Orthophosphate exhibits substantially higher concentrations in Cluster 1 than in either groundwater or the other two clusters (Figure 2b). PO_4_^3–^ is also weakly correlated with TSS in Cluster 1 samples (Pearson’s *R* = 0.34, *p* < 0.05, data not shown).

Samples in Cluster 3 exhibit elevated specific conductance (SC) and markedly higher median concentrations of sodium and chloride compared to the other clusters (Figure 2c), consistent with the wash-off of NaCl-based road deicers and anti-icers during snowmelt or rain-on-snow events.

## Discussion

The three ion clusters identified above reflect distinct hydrologic regimes and geochemical signatures. In this section, we explore their implications for: (1) identifying potential salt sources; (2) assessing risks to aquatic life in Broad Run; and (3) management actions and monitoring strategies.

### Diagnosing Watershed Salinity Sources

The hydrologic and geochemical characteristics associated with each ion cluster provide useful information for diagnosing salt sources and biogeochemical processes in urban watersheds. In this section, we focus on how relationships among ion concentrations, including how the molar concentration of one ion changes relative to another within a cluster, can offer insights into the drivers of freshwater salinization. We use this approach to evaluate four potential sources of salinity at BL30: (1) sodium- and chloride-based road deicers, (2) potassium-based airport deicers, (3) calcium- and magnesium-based deicers, and (4) natural mineral sources associated with the local Triassic basin geology.

#### Sodium- and Chloride-Based Road Deicers

The strong molar relationship between Na and Cl across all clusters (Pearson’s Correlation *R* = 0.99, 0.98, and 0.99 for Clusters 1, 2, and 3, respectively) points to NaCl-based deicers as a plausible salt source at BL30 (Figure S9a). Cross-plotting measured sodium and chloride concentrations, we find that the resulting slopes (i.e., ΔNa^+^/ΔCl^–^, where the symbol Δ denotes change) approach the 1:1 molar ratio expected for rock salt in the following order: Cluster 2 (0.68 ± 0.04), Cluster 1 (0.83 ± 0.02), and Cluster 3 (0.92 ± 0.03) (see Note S11). The lowest slope occurs under summer baseflow conditions (Cluster 2), when deicers are not being applied and transit times through the watershed are relatively long.^43^ The highest slope occurs during winter (Cluster 3), when deicer use is more likely. These patterns are consistent with NaCl inputs along with cation exchange processes that preferentially retard sodium transport (relative to chloride), leading to observed slopes <1 across all flow and seasonal conditions.^2–45^

While elevated concentrations of sodium and chloride during baseflow (Cluster 2) may reflect a combination of natural sources (such as mineral weathering from Triassic basin sediments,^46^ see below) and legacy impacts from past deicer applications that have infiltrated to groundwater,^47^ the extremely high concentrations observed during snowmelt and rain-on-snow events (Cluster 3) are more clearly attributable to recent applications of road salt and anti-icing agents.

#### Potassium-Based Airport Deicers

A likely source of potassium in Broad Run is potassium-based deicers used at Washington Dulles International Airport, a major regional facility serving over 24 million travelers annually.^48^ Because chloride salts are corrosive to aircraft, the U.S. Federal Aviation Administration permits alternatives such as potassium acetate and potassium formate, which are widely used in the aviation industry.^49^

Unlike NaCl, these potassium-based deicers lack inorganic counterions, making ion slope analysis infeasible given the set of ions measured here. However, useful information can still be gleaned from the clusters. For example, a possible alternative (nondeicer) source of potassium is the widely used fertilizer, KCl. However, the highest potassium concentrations at BL30 are associated with snowmelt and rain-on-snow events (Cluster 3, Figure 2c) when fertilizer use would be extremely unlikely. As noted earlier, potassium does not exhibit the dilution response seen for most other ions. While median K^+^ concentrations are highest in Cluster 3, they remain elevated (relative to groundwater) in both Clusters 1 and 2 (Figure 2c).

Two of the airport’s four runways drain into Broad Run, both directly through a stormwater conveyance system and indirectly via a detention pond (Figure S1). The elevated potassium concentrations observed in Cluster 3 would be consistent with inputs from the first flow path during snowmelt or rain-on-snow events. In contrast, the persistently high values in Clusters 1 and 2 are more consistent with the gradual release of potassium from the airport detention pond.

#### Calcium- and Magnesium-Based Deicers

Calcium and magnesium are active ingredients in several anti-icing products, including calcium chloride, magnesium chloride, and calcium magnesium acetate (CMA).^21,50^ Calcium and magnesium from the first two deicers would be expected to covary with chloride in a 1:2 molar ratio. However, Pearson correlation coefficients between Ca^2+^ and Cl^–^ and between Mg^2+^ and Cl^–^ are low (*R* < 0.52) across all clusters, and the corresponding slopes are not significantly different from zero (Figures S9b,c).

CMA (Mg_2_Ca­(OAc)_6_) would be expected to yield a 2:1 molar increase in magnesium relative to calcium.^51^ However, while Mg^2+^ and Ca^2+^ are strongly correlated at BL30 (*R* ≥ 0.96), the observed Mg:Ca slope is approximately 0.4 (1:2.5), inconsistent with the molecular formula of CMA (Figure S9d). These patterns suggest that calcium and magnesium are not primarily derived from deicers, pointing instead to geogenic sources consistent with the local Triassic basin geology discussed next.

#### Triassic Basin Sediments

Across all clusters, calcium and magnesium are strongly correlated with sulfate (*R* > 0.85) and bicarbonate (*R* > 0.77) (Figure S10a–d). The inferred slopes (ΔCa^2+^/ΔSO_4_^2–^ = 3.74 to 5.73, ΔCa^2+^/ΔHCO_3_^–^ = 0.40 to 0.55, ΔMg^2+^/ΔSO_4_^2–^ = 1.9 to 2.5, and ΔMg^2+^/ΔHCO_3_^–^ = 0.17 to 0.25) are similar to the corresponding median (4.9, 0.42, 3.7, 0.32, respectively) and average (5.4, 0.50, 4.1, 0.38, respectively) ratios of ion concentrations measured in the local groundwater. Further, across all three clusters, the magnesium-to-calcium ratio measured in groundwater (both median and average equal to 0.76) is close to the 1:2.5 ratio inferred for the stream (ΔMg^2+^/ΔCa^2+^ = 0.43 to 0.45) (Figure S9d). These results are also consistent with USGS groundwater data from nearby Fairfax County,^46,52,53^ which indicate that groundwater in the underlying Triassic basin sediments is predominantly calcium–magnesium type, with elevated sulfate concentrations likely derived from gypsum in the rock matrix.^54^

### Diagnosing Potential Salinity Stressors to Aquatic Life in Streams

The three ion clusters can also be linked to statewide thresholds for aquatic life stress, providing a framework for identifying likely targets for management action.^15,55,56^

The probability that chloride, sodium, and specific conductance (SC) measurements at BL30 pose stress to aquatic life ranges from low to high in Cluster 1, medium to high in Cluster 2, and consistently high in Cluster 3 (Figure 3a). Across all clusters, potassium concentrations correspond to a medium probability of stress.

**Figure 3:**
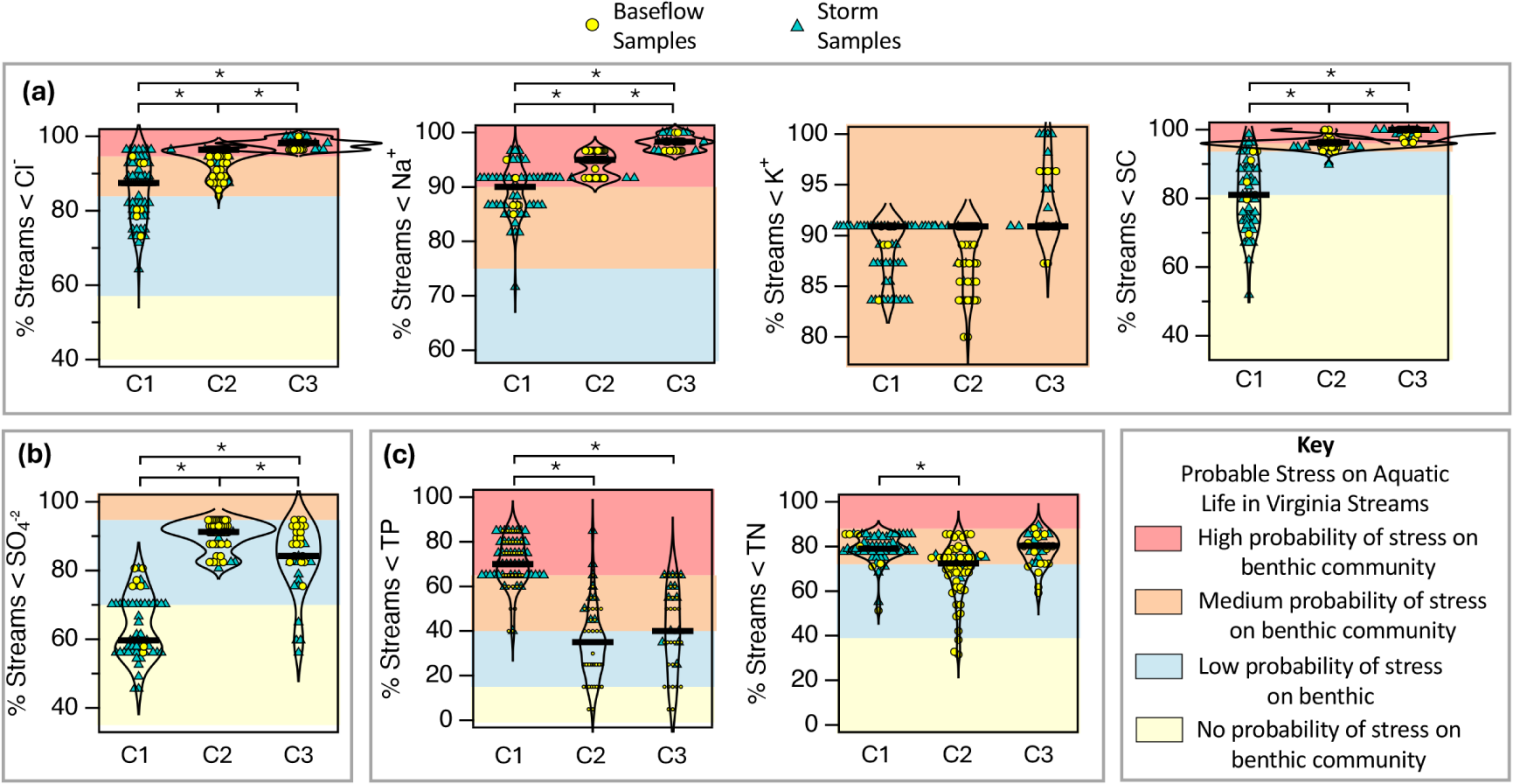
Contextualizing water quality in Broad Run and inferred probability of stress to aquatic life by cluster (C1 = Cluster 1 (summer storms), C2 = Cluster 2 (baseflow), and C3 = Cluster 3 (snowmelt and rain-on-snow events)). (a) Percentile ranking of chloride, sodium, potassium, and SC levels measured in samples collected from Broad Run (yellow circles or green triangles) relative to corresponding chloride, sodium, potassium, and SC levels measured by VDEQ in Northern Piedmont Ecoregion streams (vertical axis). Color in each plot indicates the inferred probability of stress to aquatic life (see the color key and text). The percentile rankings and inferred probabilities for aquatic life stress are highest for Broad Run samples collected during snowmelt or rain-on-snow events (Cluster 3). (b) Percentiles and inferred probability of stress on aquatic life for sulfate measurements on Broad Run. (c) Percentiles and inferred probability of stress on aquatic life for nutrients TP and TN.

Sulfate, despite being elevated during baseflow (Cluster 2), is associated with mostly low or no probability of aquatic life stress (Figure 3b). TP, in contrast, poses a high probability of stress during summer storm events (Cluster 1; Figure 3c). Because TP is highly correlated with both TSS (Pearson’s *R* = 0.86, *p* < 0.05) and flow (*R* = 0.62, *p* < 0.05), the ultimate cause of risk to benthic invertebrate assemblages is likely habitat disruption and sediment erosion associated with high-flow events.^57^ TN concentrations fall mostly in the low to medium risk range across all clusters (Figure 3c).

Together, these results point to three primary risk scenarios for salt-sensitive macroinvertebrates such as mayflies, stoneflies, and caddisflies, particularly during vulnerable life stages:^14,15,58,59^ (1) high phosphorus concentrations and associated sediment mobilization and benthic habitat disturbance during summer storms (Cluster 1); (2) somewhat elevated chloride, sodium, and SC under baseflow conditions (Cluster 2), perhaps associated with legacy deicer use; and (3) very high levels of chloride, sodium, potassium, and SC during snowmelt and rain-on-snow events associated with active deicer use (Cluster 3). These findings are consistent with low biotic and Stream Condition Index (SCI) scores in urban streams in the region,^15,60^ including Accotink Creek in neighboring Fairfax County, for which chlorides from deicers have been identified as a primary stressor.^61^

Low SCI scores in Broad Run have resulted in its recent listing as impaired for aquatic life uses by VDEQ.^62,63^ Upon completion of the required stressor identification study, salinity is likely to again be identified as a primary aquatic life stressor in this urbanized watershed, for which management actions will be required. By directly relating our ion measurements to VDEQ’s aquatic life stress thresholds, the results presented here should help local jurisdictions, state agencies, and natural resource managers prioritize monitoring efforts, particularly under resource constraints, and focus on the ion mixtures and hydrologic regimes most likely to affect the biological integrity of Broad Run.^64^

### Watershed-Based Integrated Management

The statistical pattern recognition tools employed here offer a new “tool-in-the-toolbox” for designing targeted streamwater quality management actions. Further, these tools provide a statistically grounded alternative to subjective sample classification based on presumed hydrologic regimes (e.g., see Figure S12) and may be particularly useful in settings where discharge data are unavailable or uncertain. Our findings suggest at least three key opportunities for improved streamwater quality management at BL30.

#### Targeting Seasonal Flow Regimes for Monitoring and Nutrient Reduction

Clusters defined by patterns in stream ion chemistry reveal when and how risks to aquatic life and downstream uses arise. For example, phosphorus concentrations pose risks to aquatic life during summer storms (Cluster 1), suggesting that storm-driven erosion and sediment transport are key phosphorus sources.^65^ Riparian buffers, street sweeping, and stormwater controls such as bioretention or filtration basins could be deployed to reduce particulate and particle-bound phosphorus loads.^65^

#### Confirming Source Hypotheses with Targeted Monitoring

The elevated potassium concentrations observed in Cluster 3 (snowmelt events) and persistent potassium signals in Clusters 1 and 2 suggest both event-driven and delayed contributions from airport deicers. To confirm this source attribution, follow-up grab sampling immediately downstream of the airport, timed with winter deicing events and summer pond drawdowns, could help trace potassium acetate/formate usage. Similarly, a focused winter sampling campaign at road-adjacent outfalls could help verify whether NaCl-based deicers explain the elevated chloride and specific conductance values in Cluster 3.

#### Supporting Smart Growth and Best Management Practices

By resolving overlapping sources through ion ratios and covariance patterns, our results can also inform efforts to reduce salt inputs to streams via smart growth strategies and best management practices (BMPs).

Riparian and conservation zones are a smart growth strategy that mitigate salt pollution,^22,66^ attenuating ions through plant uptake, ion exchange, and biogeochemical cycling. Their effectiveness depends on factors such as buffer width, vegetation type, and site-specific conditions.^67^ Our ion clusters reveal that ion and nutrient concentrations and their implied risks to aquatic life vary seasonally. For instance, phosphorus concentrations are highest during summer storms (Cluster 1), whereas ions linked to deicers and anti-icers peak during winter snowmelt and rain-on-snow events (Cluster 3). These patterns can inform hypotheses about dominant nutrient and salt attenuation mechanisms in riparian and conservation zones—for example, plant uptake of phosphorus during the growing season and soil-mediated ion exchange during winter months—for future evaluation.

While traditional BMPs were designed primarily to reduce peak flows,^68^ modern green infrastructure—including green roofs, permeable pavements, bioswales, and bioinfiltration systems—can also reduce sediment, phosphorus, and bacterial loading during storms.^69,70^

The next generation of these systems could also target dissolved salts, for example, by incorporating ion-exchange media or real-time monitoring and control systems to capture salt in runoff from roads and parking lots during deicer washoff events (Cluster 3).^71–73^ For example, Long et al.^74^ recently reported that cattails in a standard-sized highway detention basin (2000–3000 m^2^) could remove up to 200 kg of deicer-associated Cl^–^ per year. Alternatively, high-frequency specific conductance sensors could trigger retention of salt-rich runoff when conductivity spikes,^75–77^ followed by longer-term treatment (e.g., phytoremediation^74^) or controlled disposal (e.g., pump-and-haul). Retained runoff could also be gradually released during subsequent deicer-free storms, effectively diluting the water of Cluster 3 with the water of Cluster 1.

Information gleaned from the ion clusters could also help local jurisdictions select locally tailored deicing formulas likely to have the least impact on aquatic life, for example, by mimicking ion mixtures already contributed to streams by the local geology. However, for these bespoke deicers to be effective, the applied concentrations of individual ions would still need to be high enough to achieve a desired eutectic temperature.^78^

While further research is needed to evaluate the feasibility, scalability, costs, and potential unintended consequences of these strategies,^5,70,74,79^ the ion covariance patterns identified through ordination and clustering offer a powerful starting point for generating hypotheses and guiding the design of next-generation solutions to reverse freshwater salinization.

## Supplementary Material



## Notes

Notes S1–S13, including figures and tables, provide additional details on watershed characteristics, water quality analyses, statistical methods, hydrologic and ecological data, and concentration-discharge relationships (PDF)
