# Supplementary material for: Ion Clusters Reveal the Sources, Impacts, and Drivers of Freshwater Salinization
Source: Environ Sci Technol. 2025 Jun 16;59(27):14053–62. doi: 10.1021/acs.est.5c04512 (PMC12269075; doi:10.1021/acs.est.5c04512)
Supplement: Supplementary file 1 [file es5c04512_si_001.pdf]

# Supporting Information for *Ion Clusters Reveal the Sources, Impacts, and Drivers of Freshwater Salinization*

Diver E. Marin, Stanley B. Grant,* Shantanu V. Bhide, Megan A. Rippy, Jesus D. Gomez-Velez, Robert N. Brent, Sujay S. Kaushal, Harold Post, Sydney Shelton, Shalini Misra, Erin R. Hotchkiss, Ahmed Monofy, Dongmei Alvi, Bradley Schmitz, Shannon Curtis, Christina C. Davis, Peter Vikesland, and Admin Husic

E-mail: stanleyg@vt.edu

Summary of content: 20 pages, 6 tables and 11 figures

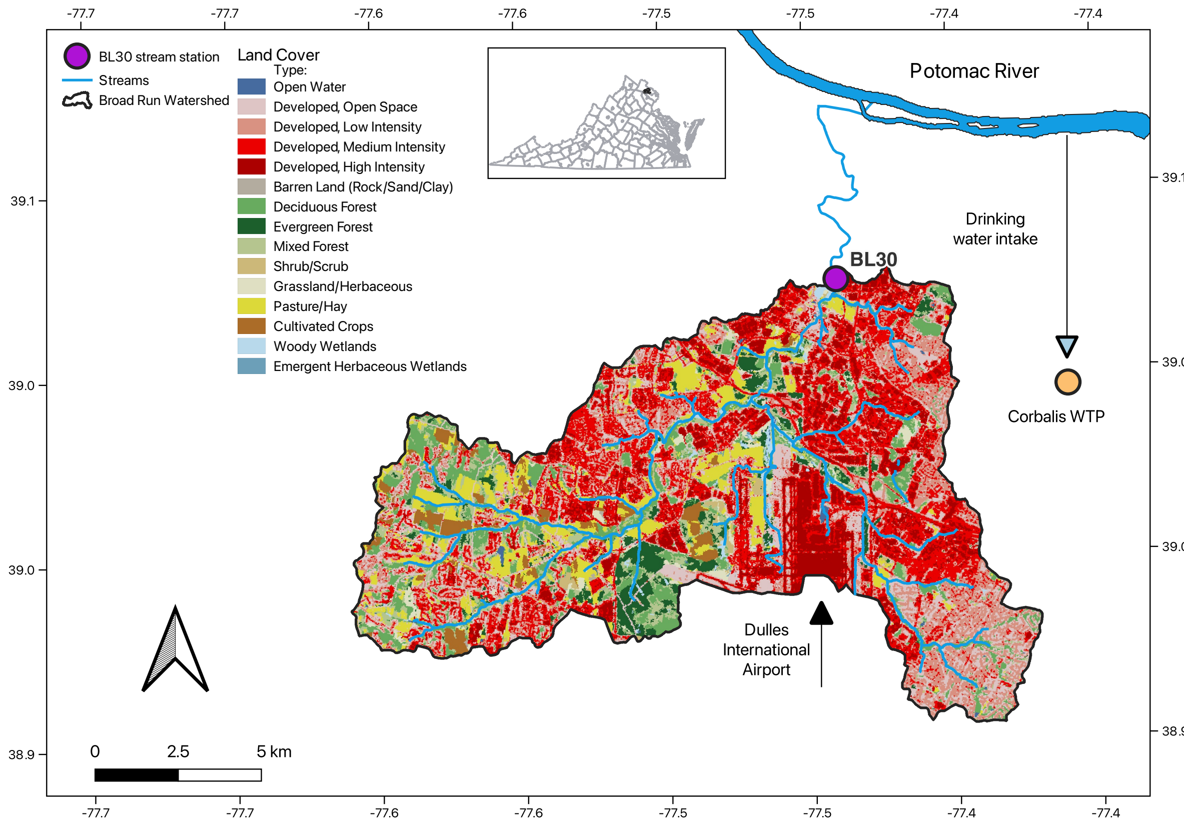


Figure S1: Broad Run drains a highly urbanized 152 km$^2$ portion of Loudoun County, Northern Virginia, USA (shown in the inset map). Our 3-year study analyzes hydrology and water quality measurements at stream station BL30 (39.024°N, 77.439°W), shown as a purple filled circle at the north-central edge of the delineated drainage. The Washington Dulles International Airport, which serves over 24 million travelers per year, is visible in the south-central portion of the drainage. The orange circle is the approximate location of the Corbalis drinking water treatment plant, which serves a population of over 1 million people. The Corbalis raw water intake is located approximately 10 km downstream of the confluence of Broad Run and the Potomac River.

Table S1: Land cover types in the portion of the Broad Run watershed that drains to BL30.

| **Cover type** | **Area ( $km^2$)** | **Percentage (%)** |
|---|---|---|
| Developed, Medium Intensity | 34.2 | 22.4 |
| Developed, Low Intensity | 28.7 | 18.8 |
| Developed, Open Space | 20.6 | 13.5 |
| Developed, High Intensity | 16.8 | 11.0 |
| Deciduous Forest | 14.0 | 9.21 |
| Pasture/Hay | 12.6 | 8.28 |
| Mixed Forest | 8.19 | 5.37 |
| Evergreen Forest | 6.33 | 4.15 |
| Cultivated Crops | 4.06 | 2.66 |
| Grassland/Herbaceous | 2.60 | 1.71 |
| Woody Wetlands | 1.97 | 1.29 |
| Shrub/Scrub | 1.25 | 0.818 |
| Open Water | 0.473 | 0.311 |
| Barren Land | 0.428 | 0.281 |
| Emergent Herbaceous Wetlands | 0.192 | 0.126 |

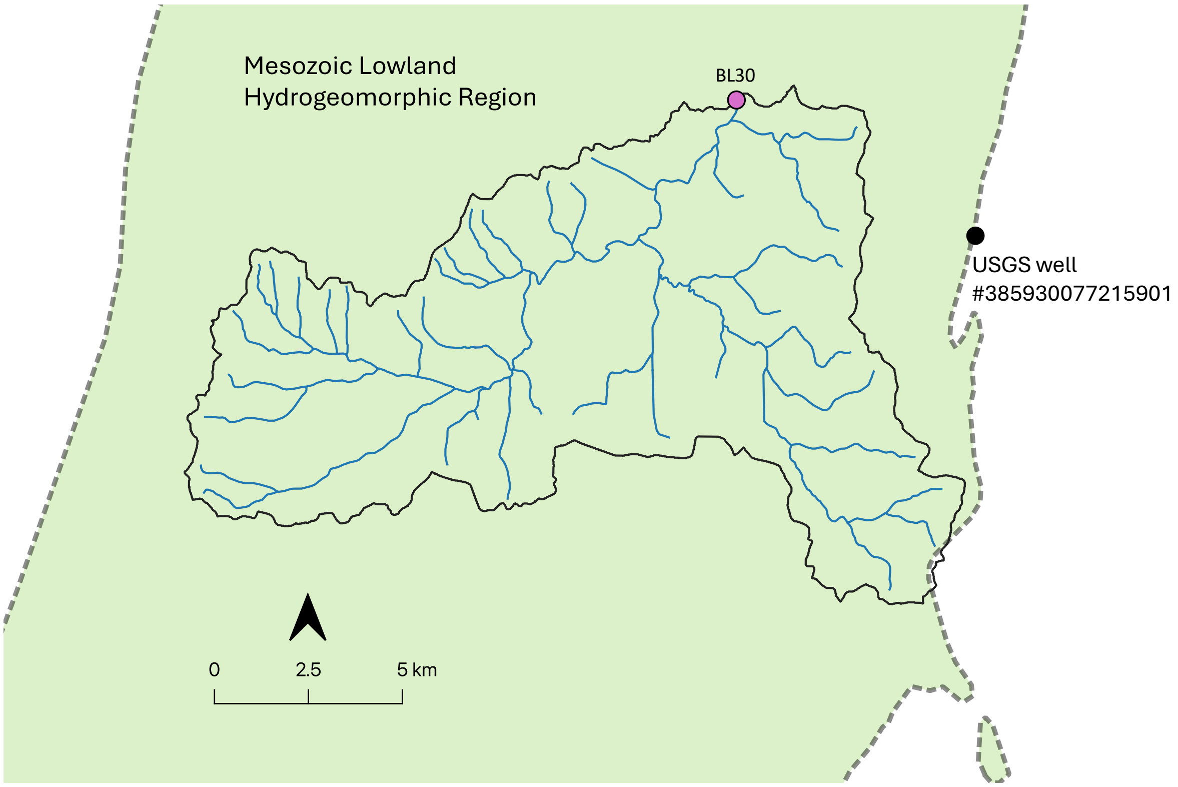


Figure S2: The location of the USGS groundwater well, relative to the BL30 monitoring station on Broad Run. The groundwater well and BL30 drainage area are located within the Mesozoic Lowland (Triassic Basin) hydrogeomorphic region of Virginia.

# Note S1: Watershed LULC and Geology

Land Use and Land Cover data were retrieved from the National Land Cover Dataset [1] and are shown in Figure S1 and summarized in Tables S1 and S2. The watershed lies within Virginia's Mesozoic Lowland Hydrogeomorphic Region, which is underlain primarily by siltstone, shale, sandstone, diabase and basalt rock types (Figures S2 and S3).

Table S2: Grouped land cover types within Broad Run watershed.

| Cover type | Area ( $km^2$) | Percentage |
|---|---|---|
| Developed | 100.25 | 65.78 |
| Forest | 28.54 | 18.73 |
| Cultivated | 16.67 | 10.94 |
| Grassland/Herbaceous | 2.60 | 1.70 |
| Wetlands | 2.16 | 1.41 |
| Shrub/Scrub | 1.24 | 0.81 |
| Open water | 0.47 | 0.31 |
| Barren land | 0.42 | 0.28 |

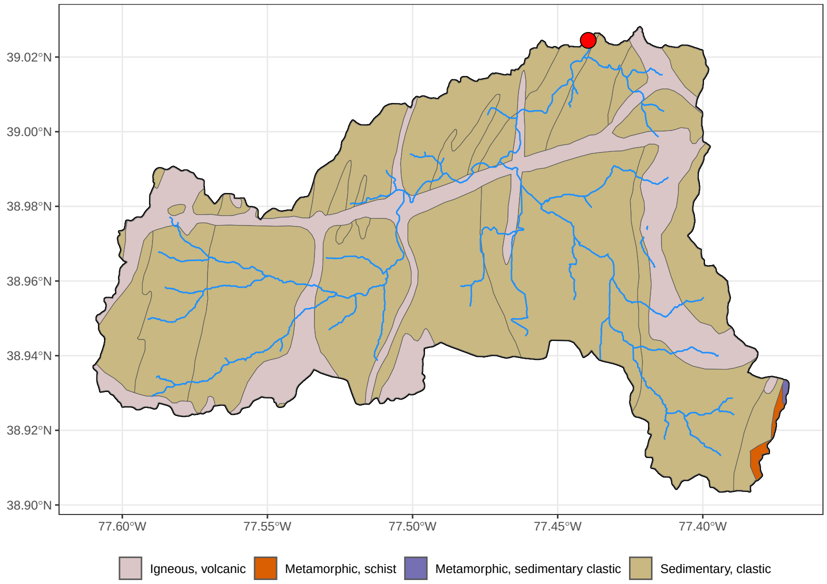


Figure S3: Geology outcrops within the portion of the Broad Run watershed that drains to BL30. [2]

# Note S2: Sample Collection and Field Measurements

Water samples were collected from station BL30 during baseflow and storm events as follows.

*Base Flow Sampling.* Baseflow grab samples were collected every other week during dry weather periods. During each baseflow sampling event, two grab samples were collected from the stream, one for dissolved ions and another for nutrient analysis. The grab sample for dissolved ions was filtered in the field through a 0.45 micron syringe filter (Filtrous Lab, 30 mm GFP filters). Stream water temperature, specific conductance, and pH were measured onsite with a multimeter (YSI Multimeter, ProDSS), which was calibrated prior to each sampling event. Stream water total alkalinity was measured onsite by sulfuric acid titration (Hach Digital Titrator). Field measurements of pH and total alkalinity were used to estimate bicarbonate concentration in each sample ($HCO_3^-$)[3] (see Note S4). For safety reasons, no field measurements were conducted during storm events. All samples were transported to the Occoquan Watershed Monitoring Laboratory on ice within 4 h for further processing. A total of 79 baseflow samples were collected and analyzed over the three-year study.

*Storm Flow Samples.* Automated flow-weighted composite samples of storm events were triggered when the rate of increase in stream stage exceeded $3.3\times10^{-5}$ feet per minute. Once triggered, a Manning Portable Vacuum Sampler (Model VST3a) was programmed to accumulate 200 mL of sample volume for every 1 million cubic feet of stream flow. For safety reasons no onsite measurements (e.g., for pH, temperature, specific conductance, or total alkalinity, see above) were collected during storm sampling events. Composite samples were transported to the Occoquan Watershed Monitoring Laboratory on ice within one day following the end of a storm. At the lab, 25 mL of the composite sample was filtered through a 0.45 micron syringe filter (Filtrous Lab, 30 mm GFP filters) for dissolved ion analysis. A total of 74 storm samples were collected over the three-year study.

## Note S3: Laboratory Analysis

Within 1 day of arriving at the lab, the pre-filtered baseflow and storm samples (see above) were analyzed for dissolved ions ($K^+$, $Na^+$, $Cl^-$, $SO_4^{2-}$, $Ca^{2+}$, $Mg^{2+}$) using ion chromatography (Dionex, ICS 5000) following ASTM D6919-09 and Standard Method 4110 B-2011.

The unfiltered baseflow and storm samples were analyzed for total and dissolved nutrients as follows.

For total nutrients, 10 mL unfiltered sample was immediately persulfate digested, stored at 4°C, and analyzed within 28 days for total nitrogen (TN, the sum of dissolved inorganic N, dissolved organic N, and particulate N species) and total phosphorus (TP, the sum of dissolved inorganic phosphorus, dissolved organic phosphorus, and particulate phosphorus) using an Astoria Pacific Model 411S Autoanalyzer with a 307 Detector (Standard Method 4500-P J-2011).

For dissolved nutrients, immediately upon arrival at the lab, approximately 200 mL of the unfiltered field sample was filtered through glass microfiber filters (Whatman, 934-AH). The filtrate was stored at -20°C and, within 28 days of arrival, analyzed for dissolved nitrate plus nitrite ($NO_3^-/NO_2^- = NO_3^- + NO_2^-$) and orthophosphate ($PO_4^{-3}$) using an Astoria Pacific, Model 311 Autoanalyzer with a 305D Detector (Standard Methods 4500-NO3- F-2011, 4500-P F-2011).

All analyses were conducted in accordance with the Occoquan Watershed Monitoring Laboratory's Virginia Environmental Laboratory Accreditation Program (VELAP #460026). The lower-limit of detection (LOD) for each analyte is indicated in Table S3.

Table S3: List of constituents measured in this study and associated LODs.

| **Chemical measures** | **Units** | **Limit of detection** |
|---|---|---|
| Chloride | mg/L | 5.0 mg/L |
| Magnesium | mg/L | 0.5 mg/L |
| Calcium | mg/L | 1.5 mg/L |
| Sulfate | mg/L | 5.0 mg/L |
| Bicarbonate | NA | NA |
| Sodium | mg/L | 1.5 mg/L |
| Potassium | mg/L | 1.0 mg/L |
| Total Alkalinity | mg/L as $CaCO_3$ | 0.1 mg/L as $CaCO_3$ |
| Total Phosphorus | mg/L | 0.01 mg/L |
| Total Nitrogen | mg/L | 0.25 mg/L |
| Specific Conductance | $\mu$S/cm | NA |
| Dissolved Orthophosphate | mg/L | 0.01 mg/L |
| Ammonium | mg/L | 0.01 mg/L |
| Oxidized Nitrogen Species | mg/L | 0.01 mg/L |

# Note S4: Bicarbonate Ion Concentrations

Bicarbonate ion concentrations were estimated using two different approaches, one approach for baseflow samples and another for storm samples. Bicarbonate concentrations in baseflow samples were estimated directly from measured total alkalinity expressed in units of mg/L as $CaCO_3$; over the pH range (6.7 to 8.1) measured in baseflow samples, most of the total alkalinity is in the form of bicarbonate. However, this approach could not be used to estimate bicarbonate ion concentrations in storm samples, because safety concerns precluded field measurements of total alkalinity during storms (see Note S2).

Instead, bicarbonate ion concentrations in storm samples were imputed using a regularized iterative PCA method that identifies dominant water quality patterns across the entire dataset (baseflow + storm samples, $N$ = 153) and "fills in" missing values based on the dominant patterns identified (see Methods Section in the main text for details). By this approach, missing bicarbonate ion concentrations were imputed for all 74 storm samples.

The accuracy of the imputed bicarbonate concentrations was checked in two ways: (1) by assessing the charge balance error (CBE) associated with measured and imputed ion

concentrations in each storm sample (see Note S5); and (2) by comparing to bicarbonate concentrations estimated using a geochemical approach, in which all unbalanced (anionic) charge is attributed to bicarbonate. Bicarbonate concentrations estimated by the latter approach are highly correlated with bicarbonate concentrations estimated by the regularized iterative PCA method (Pearson Correlation, $R$ =0.84) and fall close to, but consistently above (PBIAS=13.8%), the 1:1 line (Figure S4); note that the outlier in the cross-plot corresponds to a sample that was ultimately removed (i.e., not included in subsequent analyses) because it did not meet the requirement that absolute value of the CBE should be less than 10% (see Note S5).

This result—that bicarbonate concentrations estimated by charge balance are strongly correlated but biased high relative to bicarbonate concentrations estimated by the regularized iterative PCA method—is reasonable given that unquantified organic acids in our storm samples likely also contribute negative charge.[4] Taken together, these results provide strong support for the accuracy of bicarbonate concentrations imputed by the regularized iterative PCA method.

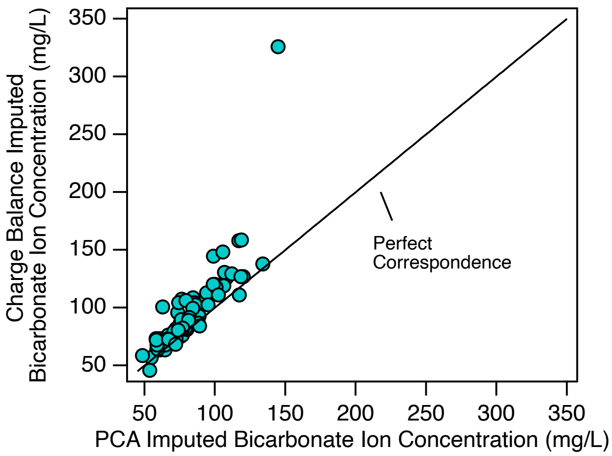


Figure S4: A comparison of bicarbonate concentrations imputed for storm samples based on either charge balance (vertical axis) or regularized iterative PCA (horizontal axis). The bicarbonate concentrations estimated by these two approaches are highly correlated, although values estimated using the geochemical approach are consistently biased high, likely due to the presence of unquantified organic acids in the storm samples.

# Note S5. Electroneutrality analysis

The measured and imputed ion concentrations in each baseflow and storm sample were checked for overall electroneutrality. Specifically, we calculated a Charge Balance Error (CBE) based on the normality, $N$, of all measured or imputed cations ($H^+$, $K^+$, $Na^+$, $Ca^{2+}$, $Mg^{2+}$, $NH_4^+$) and anions ($HCO_3^-$, $Cl^-$, $SO_4^{2-}$,$NO_3^-$/$NO_2^-$, $PO_4^{3-}$):

$$CBE(\%) = \frac{\sum N_{cation,i} - \sum N_{anion,i}}{\sum N_{cation,i} + \sum N_{anion,i}} * 100 \qquad (1)$$

Of the 153 baseflow and storm samples screened, only four (two baseflow and two storm samples) had an absolute CBE greater than 10%, a generally accepted criterion for assessing electroneutrality of ion measurements in low-ionic-strength surface waters.[4] The fact that all but two storm samples conformed to this electroneutrality criterion lends credibility to the bicarbonate concentrations imputed for all storm samples (see Note S4). The four samples with absolute CBE >10% were excluded from further analysis (i.e., they were not included in the PCA and clustering steps). The CBE for the retained 77 baseflow samples ranged from −4.37% to 6.23%, while the CBE for the retained 72 storm samples ranged from −4.2% to 7.01% (Figure S5).[4]

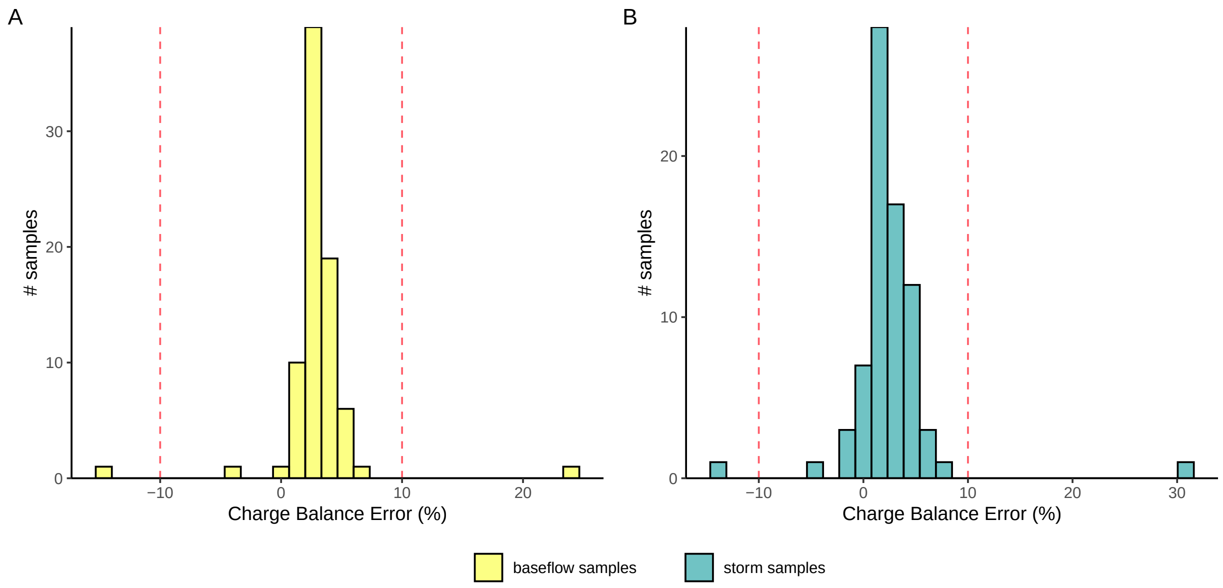


Figure S5: Charge Balance Error (CBE) calculated based on measured or imputed ion concentrations in each baseflow (left histogram) or storm (right histogram) sample. The vertical dashed red lines denote the ±10% CBE criteria typically applied to ion measurements in low ionic strength surface waters like Broad Run.

# Note S6: Environmental Data

The sources of environmental data are summarized in Table S4, while the data themselves are plotted as a time series in Figure S6 and described in more detail below.

Table S4: Environmental data included in this study

| **Variable** | **Time period** | **Source** |
|---|---|---|
| Streamflow | 2010-2023 | OWML |
| Precipitation | 2010-2023 | NOAA |
| Temperature | 2010-2023 | NOAA |
| Rainfall | 2010-2023 | HBV model |
| Snowmelt | 2010-2023 | HBV model |

*Stream Discharge.* Stream discharge was measured at station BL30 at an hourly time step by the Occoquan Watershed Monitoring Laboratory (OWML) from 2020 to 2023. These hourly discharge data were averaged to a daily time step for further analysis. Continuous

discharge data were missing on only five days, due to routine maintenance of the gauge at BL30: 2020-06-10, 2021-01-21, 2021-05-26, 2022-06-13 and 2023-02-12. These one-day long data gaps were filled using linear interpolation (zoo package in R).[5]

*Precipitation.* Daily precipitation measured at the Washington Dulles International Airport station was retrieved from NOAA. For input to the HBV model (Note S8), these daily data were extrapolated to an hourly time step using the persistence model. Trace measurements of precipitation were taken as equal to zero.

*Air Temperature.* Daily air temperature measured at the Washington Dulles International Aiport weather station was retrieved from NOAA, including daily average, minimum and maximum temperatures. For input to the HBV model (described below), these daily data were converted to an hourly time step using the package ChillR in R,[6] which generates hourly temperature records for a particular location based on minimum and maximum daily temperature, along with the location's latitude.

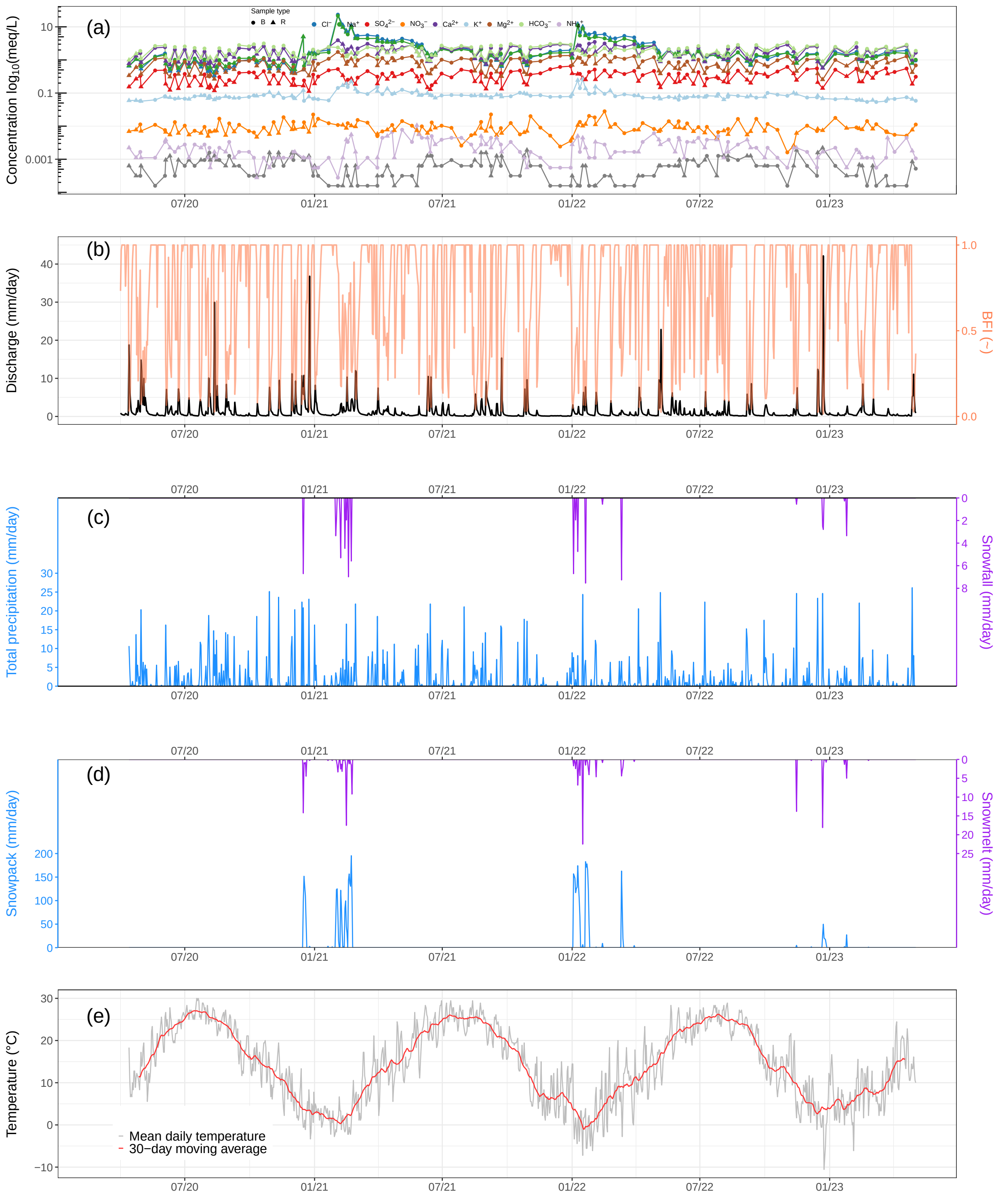


Figure S6: Daily timeseries of: a) ion measurements ($Ca^{2+}$, $Mg^{2+}$, $NH_4^+$, $K^+$, $Na^+$, $Cl^-$, $SO_4^{2-}$, $NO_3^-/NO_2^-$, $HCO_3^-$) in baseflow and stormflow samples; b) snowmelt and rainfall; c) discharge and BFI; and d) air temperature.

## Note S7: Baseflow Index (BFI)

To estimate base flow we applied a Recursive Digital Filter (RDF) to the stream discharge measurements at BL30. The RDF method isolates the high-frequency (i.e., quick flow) and low-frequency (i.e., base flow) signals from the stream discharge hydrograph using a digital filter.[7] For these calculations we used the grwat package in R, and adopted the Lyne and Hollick (LH) digital filter as follows:

$$q_{f(i)} = aq_{i-1} + \frac{1+\alpha}{2}(q_i - q(i-1)); q_{f(i)\geq 0} \tag{2}$$

$$q_{b(i)} = q_i - q_{f(i)} \tag{3}$$

Here, the subscript $i$ is the time step, $q_i$ is the total streamflow at time step i, $q_{f(i)}$ and $q_{b(i)}$ are the filter quickflow and baseflow at time step i, and $a$ is a dimensionless filter parameter which can vary from. $a \in \{0, 1\}$.[7] For our analysis we adopted the value $a = 0.925$ as recommended by Zhang et al.[8] Base Flow Index (BFI) was then computed from the ratio of the estimated baseflow and measured streamflow. Measured stream flow at BL30 is compared with baseflow estimates in Figure S7.

## Note S8: Hydrologiska Byråns Vattenbalansavdelning (HBV) Model Estimates of Snowmelt

The HBV model is a rainfall-runoff model with routines for different components of the water cycle (e.g., snow routine, soil routine, groundwater routine for response and routing, etc.)[9] The snow routine of the HBV model was used to capture (1) the partitioning of precipitation, $P(t)$, between rainfall, $r(t)$, and snowfall, and (2) the accumulation of snowpack and generation of snow melt, $s(t)$. Model parameters include (1) the threshold temperature, $T_T$

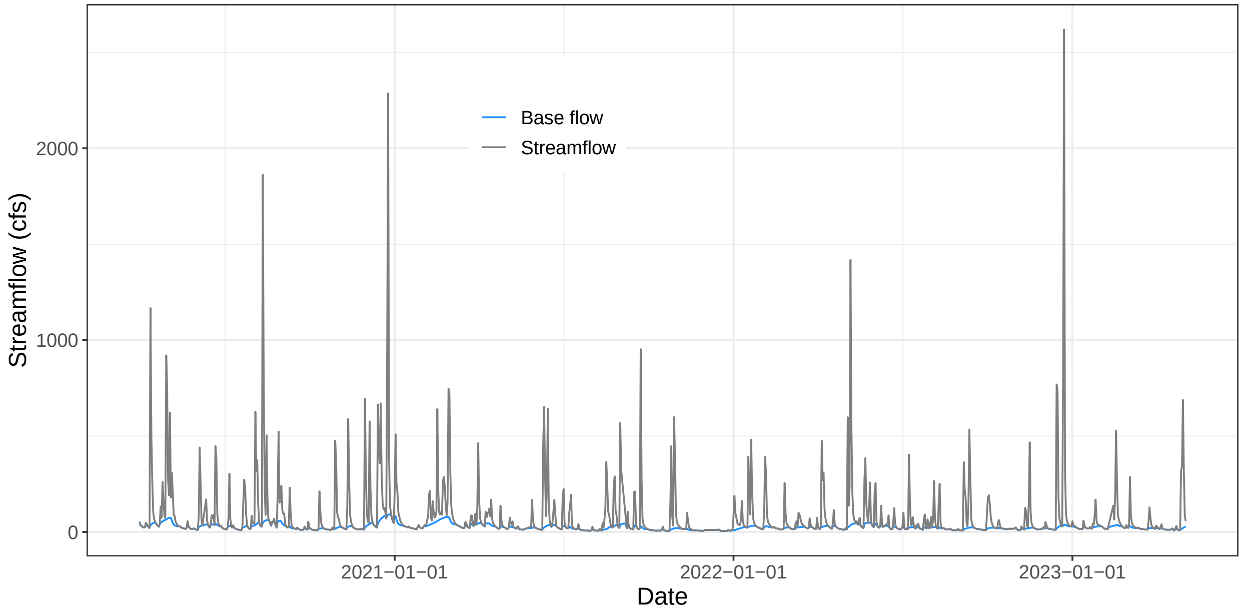


Figure S7: Measured streamflow at BL30 station compared to base flow estimated using a recursive digital filter. For the recursive filter we adopted the Lyne and Hollick method with $a = 0.925$.

(in °K), below which all precipitation is considered to fall as snow, (2) the snowfall correction factor, $SF_{cf}$, which accounts for snowfall undercatch due to wind turbulence, catchment vegetation, and other factors,[10,11] (3) the degree-day factor, $D_F$, which is a proportionality constant for estimating snow melt, (4) the water holding capacity of the snowpack, $W_H$, and (5) the refreezing coefficient, $F$, which allows for refreezing of the melted water when air temperature, $T_{air}(t)$ (in °K), falls below $T_T$. Model parameters for snow accumulation were selected to represent weather conditions in the Mid-Atlantic region of the United States (Table S5). Accumulated snow depth at Dulles International Airport, downloaded from the NOAA website (www.ncei.noaa.gov), was used to verify that the timing of HBV model-predicted snow melt events aligned with the date of zero accumulated snow depth reported at Dulles Airport after snow events. From these comparisons, we estimated that the HBV snow model accurately predicts snow melt at Dulles roughly 83% of the time.

Table S5: HBV model parameters.

| Parameter | Value |
|---|---|
| $T_T$ | 0° C |
| $SF_{cf}$ | 1.1 |
| $D_F$ | $7.29 \times 10^{-5}$ m °C$^{-1}$ h$^{-1}$ |
| $W_H$ | 0.1 |
| $F$ | 0.05 |

# Note S9: Resampling-Based Approach for Identifying Significant Principal Components

A resampling-based stopping rule was used to identify the principal components (PCs) that explained significantly more variance in the ions measurements than would be expected by chance (at the 95% confidence level) (Figure S8). The method was implemented as follows:[12] 1) Principal Component Analysis (PCA) was performed on log-transformed and Z-scored data and the eigenvalues for each principal component were saved (EIGdata); 2) Variables in the data matrix were randomized 10,000 times; 3) PCA was conducted on these randomized matrices and the eigenvalues for each principal component were saved (EIGrand); 4) percentile-based 95% confidence intervals were calculated for each principal component using the generated EIGrand values (10,000 realizations per mode); 5) PCs for which EIGdata exceeded $\geq$ the 95th percentile value of EIGrand were determined to be significant and retained as dominant dimensions.

# Note S10. Benthic Community Response Thresholds

The VDEQ state-wide ion thresholds and probability monitoring data described in the main text were compared to ion and nutrient measurements at BL30 as follows (see also[13]): (1) VDEQ's probability monitoring dataset was filtered to include only sites in the Northern Piedmont Ecoregion where BL30 is located; (2) A set of cumulative distribution functions

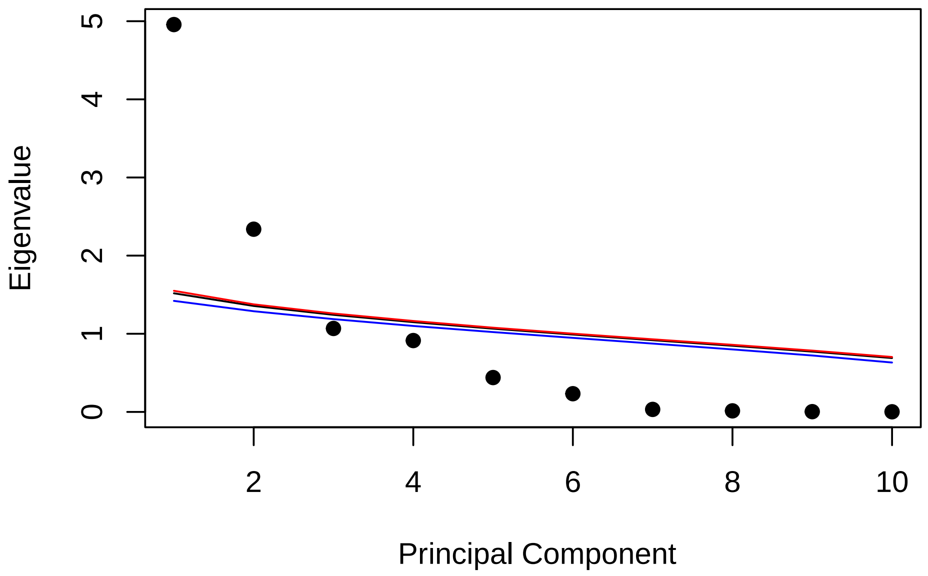


Figure S8: Principal components ($x$-axis) and associated eigenvalues ($y$-axis) for the PCA analysis conducted in this study. Also shown are the resampling-based stopping rule estimates for the eigenvalues expected by chance for a random threshold of 95%, 90%, and %50 (red, black, and blue lines, respectively). PC1 and PC2 exceed the upper 95% confidence threshold (red line) implying that they these two principal components are significant at a $p \leq 0.05$.

(CDFs) (one for each ion and nutrient) were prepared for VDEQ's stream ion and nutrient measurements in the Northern Piedmont Ecoregion; (3) using the CDFs from (2), each ion and nutrient measurement at BL30 was converted into a corresponding non-exceedance probabilities (i.e., the percentage of stream sites in the Northern Piedmont Ecoregion with that concentration or lower); and (4) results from (3) were combined with VDEQ's state-wide benthic community response thresholds, to estimate probable impact on aquatic life.

## Note S11. Ion Slopes

The salt ion concentrations measured in an urban stream likely reflect a time-varying mixture of various watershed salt sources, each with its own set of potentially unique ion ratios, along with myriad biogeochemical reactions that occur as the ions transport along flow paths through the watershed.[14] The overprinting of multiple sources and biogeochemical processes implies that ion ratios measured in the stream are unlikely to have much diagnostic value.

On the other hand, if a particular source is driving sample-to-sample variability in measured salinity, its ion ratio might manifest as a consistent molar increase in one ion relative to another; i.e., the slope obtained when the molar concentrations of the two ions are cross-plotted. For example, at BL30 we might predict that an increase in the molar concentration of sodium, $\Delta Na^+$ (where the symbol $\Delta$ denotes increase), is associated with a near equal increase in the molar concentration of chloride, $\Delta Cl^-$, given the dominant use of NaCl (in either its crystalline or brine forms) for road and parking lot deicers and anti-icers.[15]

To test this idea, we cross-plotted the molar concentrations of ion pairs commonly associated with deicers (Figure S9) and geogenic sources (Figure S10). The deicers evaluated included NaCl (Figure S9a), $CaCl_2$ (Figure S9b), $MgCl_2$ (Figure S9c), and magnesium calcium acetate ($Mg_2Ca(OAc)_6$) (Figure S9d). Geogenic sources are assumed to lead to correlated increases in the molar concentrations of magnesium and sulfate (Figure S10a), calcium and sulfate (Figure S10b), magnesium and bicarbonate (Figure S10c), and calcium

and bicarbonate (Figure S10d).

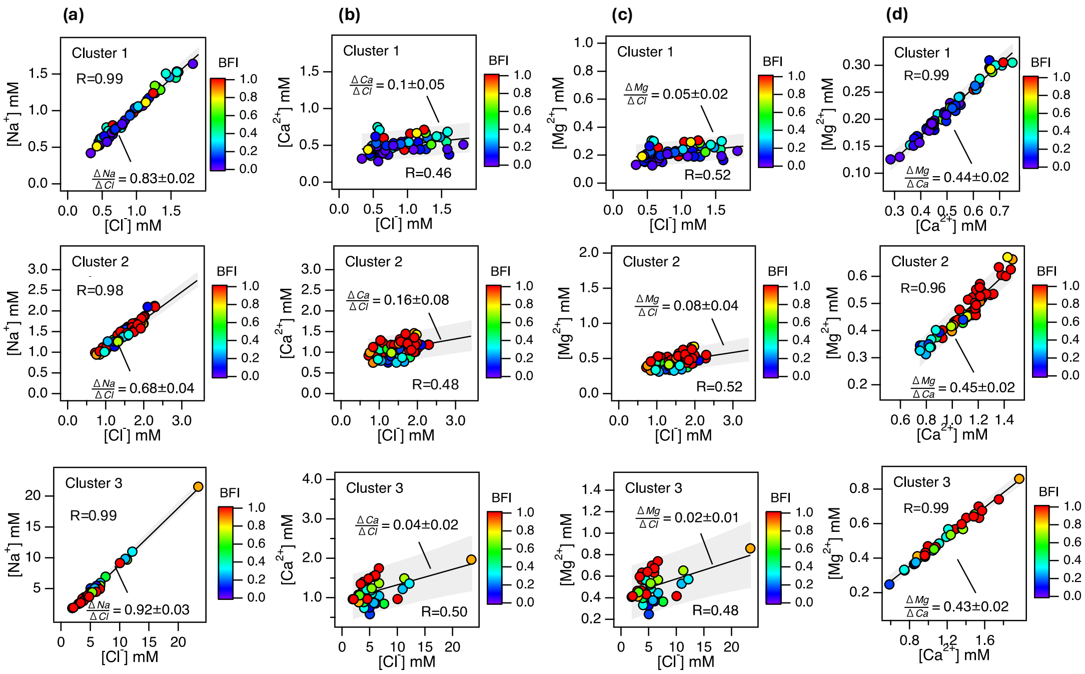


Figure S9: Cross-plots of the molar concentrations of ion pairs associate with specific deicers and anti-icers, including: (a) NaCl, (b) $CaCl_2$, (c) $MgCl_2$ and (d) $(Mg_2Ca(OAc)_6)$.

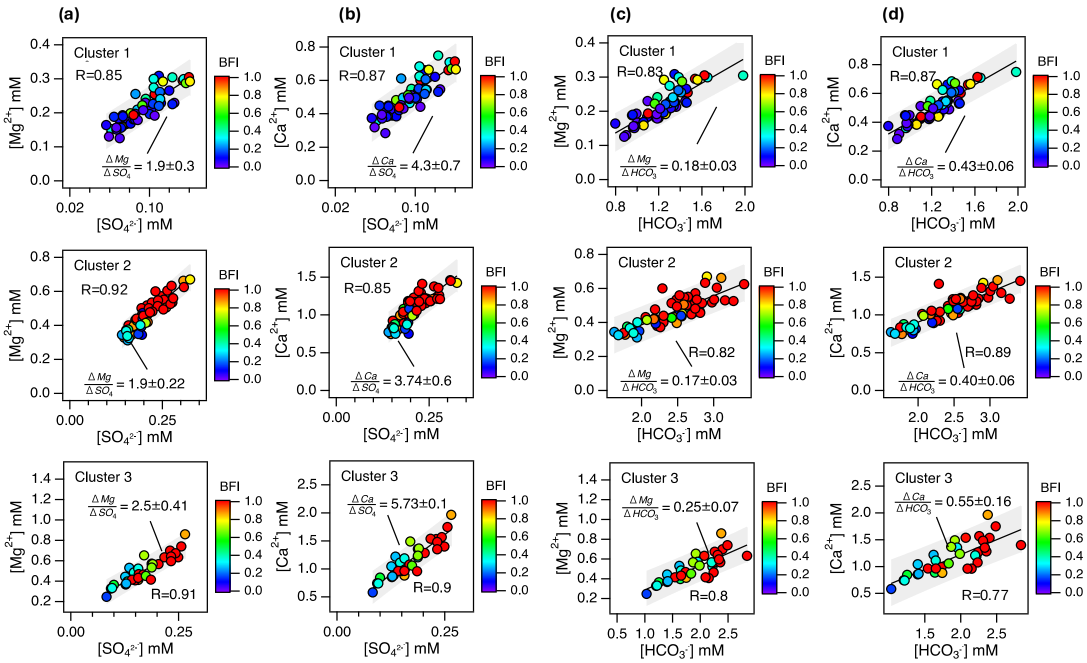


Figure S10: Cross-plots of the molar concentrations of ion pairs associate with geologic sources, including: (a) magnesium and sulfate, (b) calcium and sulfate, (c) magnesium and bicarbonate, and (d) calcium and bicarbonate.

# Note S12. Nutrient Concentrations within Clusters

The concentrations, forms, and stoichiometry of N and P measured at BL30 also vary across the three clusters. The median concentration of TP—which includes dissolved inorganic $PO_4^{3-}$ along with particulate and organic forms of P ("other-P")—is highest during summer storms (Cluster 1, Figure S11a). Cluster 1 also has elevated concentrations of $PO_4^{3-}$ (Figure 2b in the main text) and TSS (Figure S11a) consistent with the idea that summer storms mobilize particle-associated P (e.g., from fertilized residential and urban soils, pet waste, and yard waste such as leaves and grass clippings) thereby increasing the concentrations of both dissolved $PO_4^{3-}$ and other-P.[16–20] Yang et al.[16] noted that $PO_4^{3-}$ was the dominant form of P in $> 90\%$ of stormwater runoff from the residential community in Florida, while in Broad Run we find that other-P, not $PO_4^{3-}$, is the dominant form in most baseflow and stormwater samples (i.e., median $PO_4^{3-}$/TP ratios are all less than 0.5 across the three clusters, Figure

S11a).

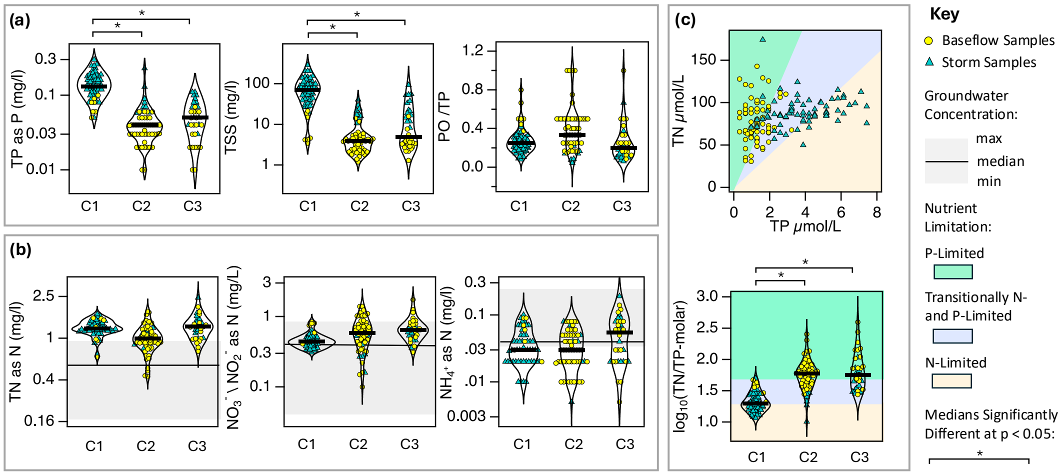


Figure S11: Breakdown by cluster of: (a) total phosphorus (TP), total suspended solids (TSS), and the fraction of TP that is dissolved orthophosphate ($PO_4^{3-}$); and (b) total nitrogen (TN), $NO_3^-/NO_2^-$ (nitrate plus nitrite), and ammonium. The black horizontal lines and gray regions for TN, $NO_3^-/NO_2^-$, and ammonium indicate the median and range of concentrations measured in the local groundwater. Groundwater concentrations of TP and TSS were not reported. (c) The stoichiometry of TN and TP in storm and baseflow samples and by cluster. The green, purple, and orange regions in (c) represent conditions under which algal growth is likely to be P-limited (TN /TP > 50), N-limited (TN/TP < 20), and transitional between P- and N-limited (20 < TN/TP < 50), where TN and TP are expressed as molar concentrations of N and P, respectively.

Most of the TN measured at BL30 is in the form of $NO_3^-/NO_2^-$, and to a much lesser extent $NH_4^+$ (compare three graphs in Figure S11b). The median concentrations of all three forms of N evaluated here (TN, $NO_3^-/NO_2^-$, $NH_4^+$) are not significantly different across the three clusters but are similar to local groundwater concentrations (Figure S11b).

Based on a global assessment of algal growth in freshwater lakes and oceans, Guildford and Hecky[21] proposed a set of stoichiometric relationships for TN and TP when algal growth is P-limited (TN/TP > 50), N-limited (TN/TP < 20), or transitional between N and P limited (20 < TN/TP < 50), where TN and TP are expressed as molar concentrations

of N and P, respectively. Similar stoichiometric N and P thresholds have been applied to stormwater runoff.[20]

Applied to our Broad Run dataset, we find that a subset of baseflow samples fall in the P-limited range, a subset of the storm samples fall in the N-limited region, and a subset of both storm and baseflow samples are transitionally P- and N-limited (top graph, Figure S11c). Roughly half of the stream samples collected during summer storms (Cluster 1) are N-limited while the other half are transitionally N- and P-limited (bottom graph, Figure S11c). On the other hand, about half of samples collected during baseflow (Cluster 2) and during winter snow-melt or rain-on-snow events (Cluster 3) are P-limited, while the other half are transitionally N- and P-limited.

# Note S13. Ion and Nutrient Concentrations vs Flow

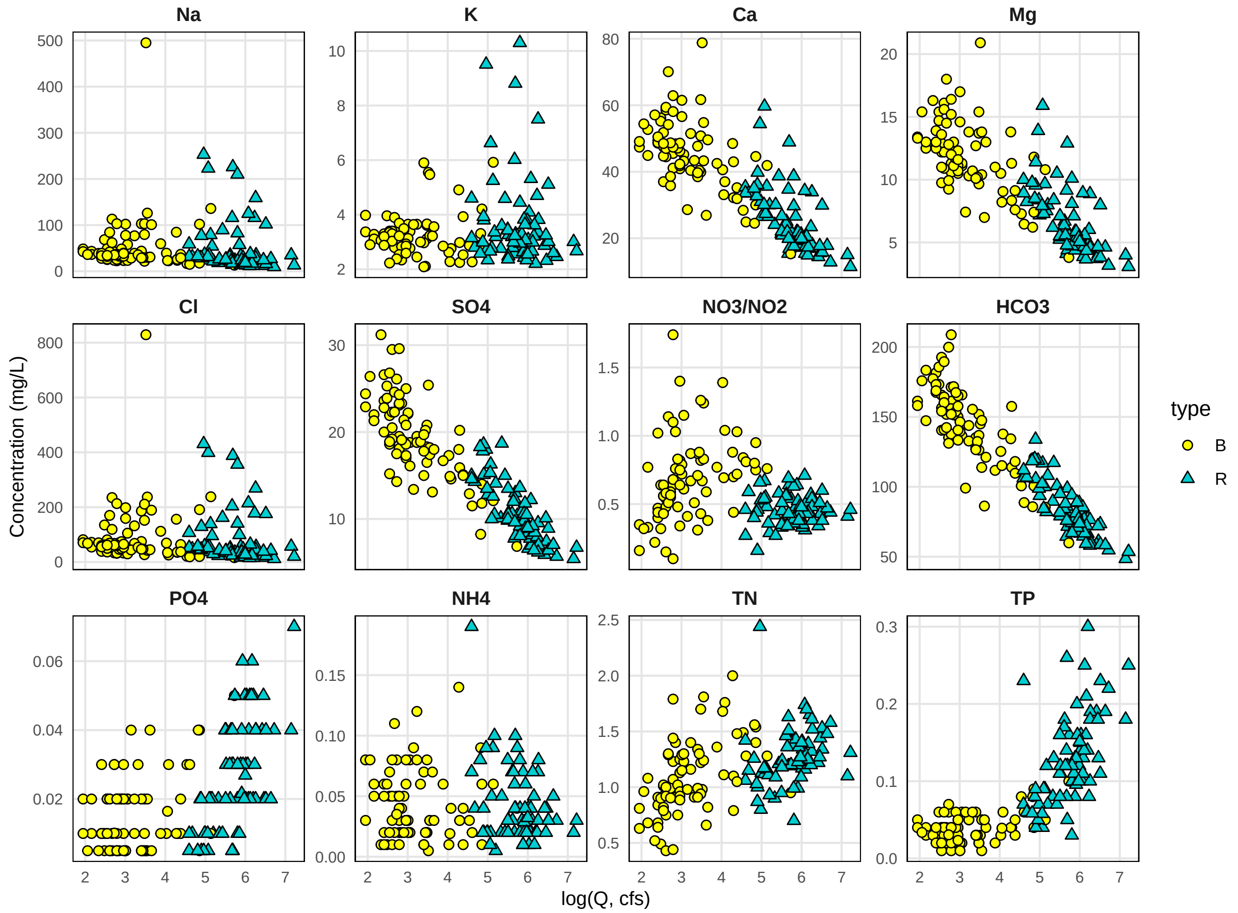


Figure S12: Concentrations of major ions and nutrients plotted against log-transformed discharge for baseflow (yellow circles) and storm flow (teal triangles) samples.

## References


(1) Dewitz;Jon, U. G. S. National Land Cover Database (NLCD) 2019 Products (ver. 2.0 June 2021). 2021.

(2) Horton, J. D. Geologic Map of Virginia. 2017; https://mrdata.usgs.gov/geology/state/.

(3) Butler, J. N. *Solubility and pH calculations: the mathematics of the simplest ionic equilibria*; Addison-Wesley Reading, MA, 1964.

(4) Katz, B. G.; Collins, J. J. *Evaluation of chemical data from selected sites in the Surface-Water Ambient Monitoring Program (SWAMP) in Florida*; US Geological Survey, 1998; Vol. 98.

(5) Zeileis, A.; Grothendieck, G. **zoo** : *S3* Infrastructure for Regular and Irregular Time Series. *Journal of Statistical Software* **2005**, *14*.

(6) Eike, L. ChillR: Statistical methods for Phenology Analysis in Temperate Fruit trees. 2020; https://CRAN.R-project.org/package=chillR.

(7) Li, L.; Maier, H. R.; Partington, D.; Lambert, M. F.; Simmons, C. T. Performance assessment and improvement of recursive digital baseflow filters for catchments with different physical characteristics and hydrological inputs. *Environmental Modelling & Software* **2014**, *54*, 39–52.

(8) Zhang, J.; Zhang, Y.; Song, J.; Cheng, L. Evaluating relative merits of four baseflow separation methods in Eastern Australia. *Journal of Hydrology* **2017**, *549*, 252–263.

(9) Merz, R.; Parajka, J.; Blöschl, G. Time stability of catchment model parameters: Implications for climate impact analyses. *Water Resources Research* **2011**, *47*, 2010WR009505.

(10) Girons Lopez, M.; Vis, M. J. P.; Jenicek, M.; Griessinger, N.; Seibert, J. Assessing the degree of detail of temperature-based snow routines for runoff modelling in mountainous areas in central Europe. *Hydrology and Earth System Sciences* **2020**, *24*, 4441–4461.

(11) Shrestha, M.; Wang, L.; Koike, T.; Tsutsui, H.; Xue, Y.; Hirabayashi, Y. Correcting basin-scale snowfall in a mountainous basin using a distributed snowmelt model and remote-sensing data. *Hydrology and Earth System Sciences* **2014**, *18*, 747–761.

(12) Peres-Neto, P. R.; Jackson, D. A.; Somers, K. M. How many principal components?

stopping rules for determining the number of non-trivial axes revisited. *Computational Statistics & Data Analysis* **2005**, *49*, 974–997.

(13) Virginia Department of Environmental Quality *Stressor Analysis in Virginia: Data Collection and Stressor Thresholds*; 2017.

(14) Shelton, S. A.; Kaushal, S. S.; Mayer, P. M.; Shatkay, R. R.; Rippy, M. A.; Grant, S. B.; Newcomer-Johnson, T. A. Salty chemical cocktails as water quality signatures: Longitudinal trends and breakpoints along different U.S. streams. *Science of The Total Environment* **2024**, *930*, 172777.

(15) Sivers, S.; Isenberg, W.; Evans, D. *Salt Management Strategy: A Toolkit to Reduce the Environmental Impacts of Winter Maintenance Practices*; 2020.

(16) Yang, Y.-Y.; Toor, G. S. Stormwater runoff driven phosphorus transport in an urban residential catchment: Implications for protecting water quality in urban watersheds. *Scientific Reports* **2018**, *8*, 11681.

(17) Hobbie, S. E.; Finlay, J. C.; Janke, B. D.; Nidzgorski, D. A.; Millet, D. B.; Baker, L. A. Contrasting nitrogen and phosphorus budgets in urban watersheds and implications for managing urban water pollution. *Proceedings of the National Academy of Sciences* **2017**, *114*, 4177–4182.

(18) Selbig, W. R. Evaluation of leaf removal as a means to reduce nutrient concentrations and loads in urban stormwater. *Science of The Total Environment* **2016**, *571*, 124–133.

(19) Bratt, A. R.; Finlay, J. C.; Hobbie, S. E.; Janke, B. D.; Worm, A. C.; Kemmitt, K. L. Contribution of Leaf Litter to Nutrient Export during Winter Months in an Urban Residential Watershed. *Environmental Science & Technology* **2017**, *51*, 3138–3147.

(20) Yang, Y.-Y.; Toor, G. S. Sources and mechanisms of nitrate and orthophosphate trans-

port in urban stormwater runoff from residential catchments. *Water Research* **2017**, *112*, 176–184.

(21) Guildford, S. J.; Hecky, R. E. Total Nitrogen, Total Phosphorus, and Nutrient Limitation in Lakes and Oceans: Is There a Common Relationship? *Limnology and Oceanography* **2000**, *45*, 1213–1223.
